# Human induced pluripotent stem cell differentiation and direct transdifferentiation into corneal epithelial-like cells

**DOI:** 10.18632/oncotarget.9791

**Published:** 2016-06-02

**Authors:** Artur Cieślar-Pobuda, Mehrdad Rafat, Viktoria Knoflach, Magdalena Skonieczna, Andrzej Hudecki, Andrzej Małecki, Elżbieta Urasińska, Seaid Ghavami, Marek J. Łos

**Affiliations:** ^1^ Stem Cell Group, Nordic EMBL Partnership, Centre for Molecular Medicine Norway (NCMM), University of Oslo, Oslo, Norway; ^2^ Institute of Automatic Control, Silesian University of Technology, Gliwice, Poland; ^3^ LinkoCare Life Sciences AB, Mjärdevi Science Park, Linköping, Sweden; ^4^ Unit of Molecular Neurobiology, Department of Medical Biochemistry & Biophysics, Karolinska Institute, Stockholm, Sweden; ^5^ Center for Biotechnology, Bioengineering and Bioinformatics, Silesian University of Technology, Gliwice, Poland; ^6^ Institute of Non-Ferrous Metals, Gliwice, Poland; ^7^ Laboratory of Molecular Biology, Faculty of Physiotherapy, The Jerzy Kukuczka Academy of Physical Education in Katowice, Katowice, Poland; ^8^ Department of Pathology, Pomeranian Medical University, Szczecin, Poland; ^9^ Department of Human Anatomy and Cell Science, College of Medicine, Faculty of Health Sciences, University of Manitoba, Winnipeg, MB, Canada

**Keywords:** corneal epithelial cells, limbal cells, transdifferentiation, Klf4, Pax6

## Abstract

The corneal epithelium is maintained by a small pool of tissue stem cells located at the limbus. Through certain injuries or diseases this pool of stem cells may get depleted. This leads to visual impairment. Standard treatment options include autologous or allogeneic limbal stem cell (LSC) transplantation, however graft rejection and chronic inflammation lowers the success rate over long time. Induced pluripotent stem (iPS) cells have opened new possibilities for treating various diseases with patient specific cells, eliminating the risk of immune rejection. In recent years, several protocols have been developed, aimed at the differentiation of iPS cells into the corneal epithelial lineage by mimicking the environmental niche of limbal stem cells. However, the risk of teratoma formation associated with the use of iPS cells hinders most applications from lab into clinics. Here we show that the differentiation of iPS cells into corneal epithelial cells results in the expression of corneal epithelial markers showing a successful differentiation, but the process is long and the level of gene expression for the pluripotency markers does not vanish completely. Therefore we set out to determine a direct transdifferentiation approach to circumvent the intermediate state of pluripotency (iPS-stage). The resulting cells, obtained by direct transdifferentiation of fibroblasts into limbal cells, exhibited corneal epithelial cell morphology and expressed corneal epithelial markers. Hence we shows for the first time a direct transdifferentiation of human dermal fibroblasts into the corneal epithelial lineage that may serve as source for corneal epithelial cells for transplantation approaches.

## INTRODUCTION

The ocular surface has two main functions; refracting light, and protecting the intraocular environment by forming an effective barrier against fluid loss and pathogen penetration. It is comprised of the corneal epithelium, limbal epithelium and conjunctival epithelium [1]. Due to the exposure of the cornea to the external environment the cells of the corneal epithelium need to be constantly replaced. The limbal epithelium is thought to contain limbal epithelial stem cells (LESCs) which upon differentiation into transient amplifying cells will move centripetal to the cornea to replenish the old cells [2]. The LESCs can be distinguished from terminally differentiated corneal epithelial cells by the expression of integrin α9, ΔNp63α and ABCG2. Corneal epithelial cells express high levels of cytokeratin 3, cytokeratin 12, involucrin and connexin 43 [3]. In certain corneal diseases, like limbal stem cell deficiency, or in cases of damages from external sources the pool of LESCs is depleted, which leads to an impairment in corneal epithelial maintenance and consequent conjunctivalization. In most cases limbal stem cell transplantation is the first choice of treatment. Hereby either autologous or allogeneic limbal epithelial stem cells [4], or expanded *ex vivo* limbal epithelial stem cells are transplanted [5], however, the risk of graft rejection is high. In addition, LSC transplantation has other downsides including risks to the contralateral healthy eye, and limited cell expansion *ex vivo*. It is also not applicable if patients have damage to both eyes, if patient's LSC deficiency has a genetic background, or in Stevens-Johnsons syndrome [6]. Furthermore, chronic inflammation negatively affects the long-term survival of transplants [7].

The discovery that adult somatic cells can be reprogrammed to an undifferentiated, pluripotent state similar to embryonic stem (ES) cells has revolutionized the concept of cell fate. Those so called iPS cells can be generated by introducing the four transcription factors *Oct4, Sox2, Klf4* and *c-myc* into adult somatic cells. Those resulting cells have the ability to differentiate into all three germ layers [8, 9]. With this method ethical concerns associated with ES-cell harvesting from embryos could be avoided, as no oocytes or embryos are used. Furthermore, patient-specific cells can be used, which makes it ideal for drug screening, disease modeling and regenerative medicine. However, pluripotent cells may form teratomas, which limits the use of iPS cells in the clinics [10–12]. Especially dangerous in this context is the use of the proto-oncogenes *c-myc* and *Klf4* in reprogramming events, hence these transcription factors should be replaced. As shown by Yu and colleagues replacing *c-myc* and *Klf4* by *Nanog* and *Lin28* also lead to sufficient reprogramming of human somatic cells [13]. Still, generating iPS cells and consequently differentiating them is time-consuming, and the efficiency of reprogramming is rather low.

Converting cells from one cell lineage to another without prior dedifferentiation into pluripotent cells would eliminate the risk of teratoma formation. This concept of lineage conversion is not new, already in 1987 Davies and colleagues have shown that introduction of just cDNA alone may convert fibroblasts into myoblasts [14]. In recent years, this field has gained new interest and it has been shown that mouse fibroblasts can be transdifferentiated into neurons [15] or cardiomyocytes [16]. Furthermore, it is a rather fast process, taking between 2-4 days to complete transdifferentiation, and it occurs with high efficiency [15, 16]. So far no protocol exists for converting fibroblasts into the limbal epithelial/corneal epithelial lineage.

**Figure 1:**
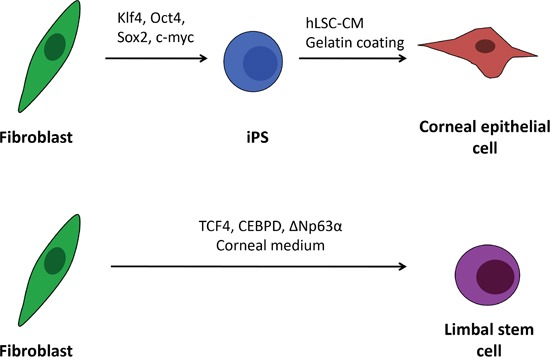
Schematic outline of the human iPS cell differentiation and direct transdifferentiation into corneal epithelial-like cells In the proposed procedures corneal epithelial cells are generated from induced pluripotent stem cell by culturing the cells on gelatine in human limbal stromal cells conditioned medium. Fibroblasts can be also directly transdifferentiated to limbal stem cells (and epithelial cells) by overexpressing three limbal specific transcription factors: TCF4, CEBPD, ΔNp63α and culturing in corneal specific medium.

In the present study, we show that it is possible to generate corneal limbal cells directly form fibroblasts. For comparison (Figure 1), we also differentiated iPS cells (reprogrammed from human dermal fibroblasts) and consequently differentiated them into cells of the corneal epithelial lineage. The expression profile for corneal epithelial-specific markers was investigated in both types of cells, and compared with human corneal epithelial cells (HCEC), that served at our experiments as a control/reference.

## RESULTS

### Generation of iPS cells from human dermal fibroblasts

**Figure 2:**
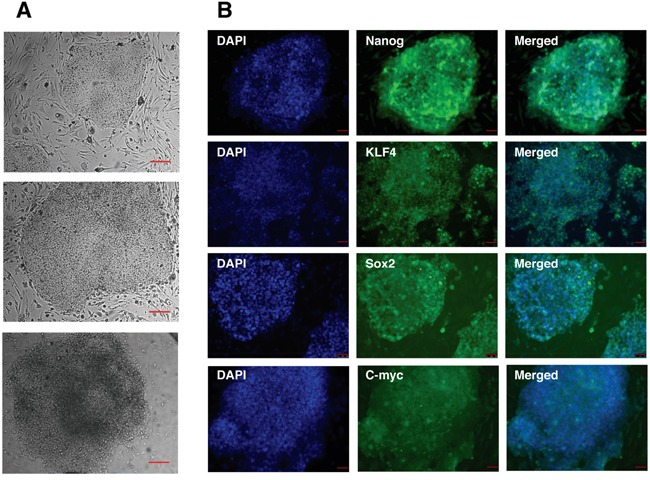
Characterization of human iPS cells generated by retroviral transduction of fibroblasts by defined factors **A.** Bright-field images of iPS cells at three, successive passages. Scale bar: 100 μm. **B.** Immunocytochemistry analysis of iPS cells for the expression of Nanog, Klf4, Sox2 and c-myc. Cells were counterstained with DAPI to visualize cell nuclei. Scale bar: 50 μm

For the generation of iPS cells human dermal fibroblasts were infected with the 4 Yamanaka factors: Oct4, Sox2, Klf4 and c-myc. After 2 weeks ES-like colonies appeared and those colonies were picked up and cultured in iPS cell culture conditions. iPS cell colonies stably maintained their iPS cell-like morphology during the whole culture period and subsequent passages (Figure 2A). To assess whether the generation of iPS cells was successful we analyzed the expression of Oct4, Sox2, Klf4, c-myc as well as the pluripotent stem cell marker Nanog by immunostaining. All iPS cell colonies analyzed expressed the pluripotency markers (Figure 2B). The ES-like morphology of the generated iPS cells, the possibility to expand iPS cell colonies and the positive immunostaining for the pluripotency markers indicate the successful reprogramming of human dermal fibroblasts to iPS cells.

### iPS cells could be differentiated to corneal epithelial cells

**Figure 3:**
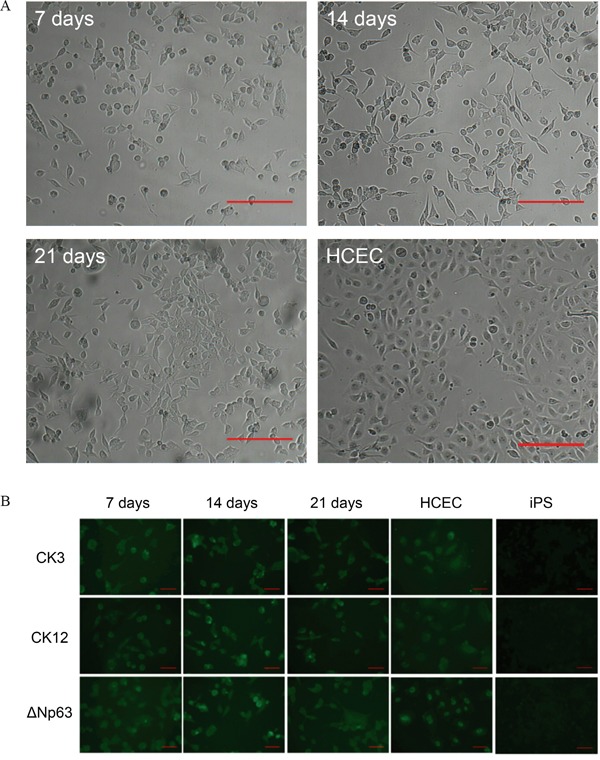
Differentiation of iPS cells to the corneal epithelial lineage **A.** Bright-field images of differentiated iPS cells at day 7 of the experiment, day 14, day 21, and HCEC as positive control. Scale bar: 200 μm. **B.** Immunocytochemistry analysis of differentiated iPS cells for the expression of CK3, CK12 & ΔNp63 at day 7, day 14 and day 21. HCEC served as positive control. Scale bar: 50 μm.

Next, we determined if our generated iPS cells could be differentiated to corneal epithelial cells by mimicking the natural environmental niche. Thereby the surface of the well was coated with gelatin and the differentiation medium was conditioned with human limbal stromal cells. The differentiation process was evaluated over a 21 days period. Already at first days of differentiation the iPS cells started to flatten and changed from a round sphere-like morphology to the cobblestone appearance typical for HCEC. This appearance did not change during the whole culture period (Figure 3A). To further examine whether our iPS cells differentiated to the corneal epithelial lineage we performed immunocytochemistry for corneal epithelial markers. The immunostaining was positive for all tested corneal epithelial markers: ΔNp63, CK3, CK12, C/EBPδ, ITF2, ABCG2 and Pax6, but to different extend. Pax6, ABCG2 and ITF2 proteins were expressed at low levels in all examined cells, whereas the expression of CK3, CK12 and ΔNp63 was stable and relatively high over the 21 days culture period (Figure 3B). To determine if the differentiation was specific to the corneal epithelial lineage, differentiated cells were also stained for CK10, a skin epithelial marker. The differentiated cells did express to some extend CK10, but the expression level was similar in HCEC stained for the same marker (Supplementary Figure S1). The expression levels for all stained proteins are summarized in Table 1.

**Table 1:** Summary of immunocytochemistry analysis

	Day 7	Day 14	Day 21	IPS cells	HCEC
**ΔNp63**	+++^a^	++	++	-	+++
**CK3**	++	+++	+++	-	++
**CK12**	++	+++	+++	-	++
**Pax6**	+	+	+	-	+
**C/EBPδ**	+++	+++	+++	++	+++
**ITF2**	++	+	+	+	-
**ABCG2**	+	+	+	-	+
**CK10**	+	+	++	-	+
**Oct4**	++	++	+++	+++	+
**Sox2**	++	++	+++	+++	+
**Klf4**	++	+	++	+++	++
**c-myc**	++	+++	+++	+++	+++
**Nanog**	+++	+++	+++	+++	+

### Immunocytochemistry reveals expression of pluripotency markers in differentiated cells

**Figure 4:**
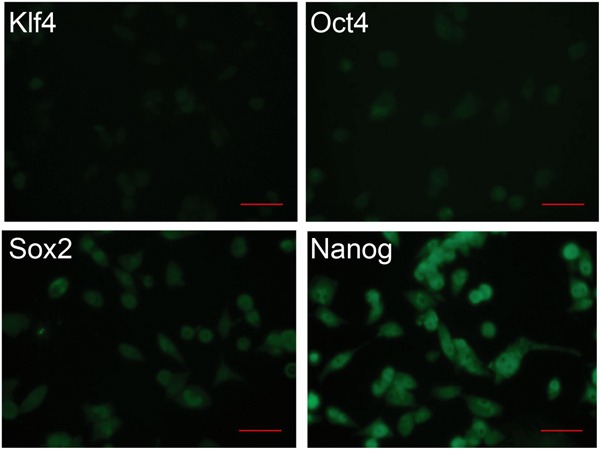
Expression of pluripotency markers in differentiated iPS cells Immunocytochemistry analysis of differentiated iPS cells for the expression of the pluripotency markers Klf4, Oct4, Sox2, and Nanog. Cells were analyzed on day 7. Scale bar: 50 μm.

One hallmark of iPS cells is their differentiation capability to give rise to cells of all three germ layers. This feature makes those cells as valuable for regenerative medicine as virtually all body organs can be grown *in vitro*, but in the same time it limits their use, as undifferentiated cells can give rise to teratomas upon transplantation. Therefore, it must be ensured that all iPS cells are differentiated and do not express any pluripotency markers anymore. Hence, we assessed the loss of iPS cell pluripotency during the differentiation process by immunostaining for Oct4, Sox2, Klf4, and Nanog. The expression levels decreased significantly only for Klf4 and partially for Oct4, whereas other markers remained at high level (Figure 4), suggesting an incomplete differentiation, which can lead upon transplantation to uncontrolled cell growth and teratomagenesis.

### RT-qPCR confirms successful differentiation of iPS cells

**Figure 5:**
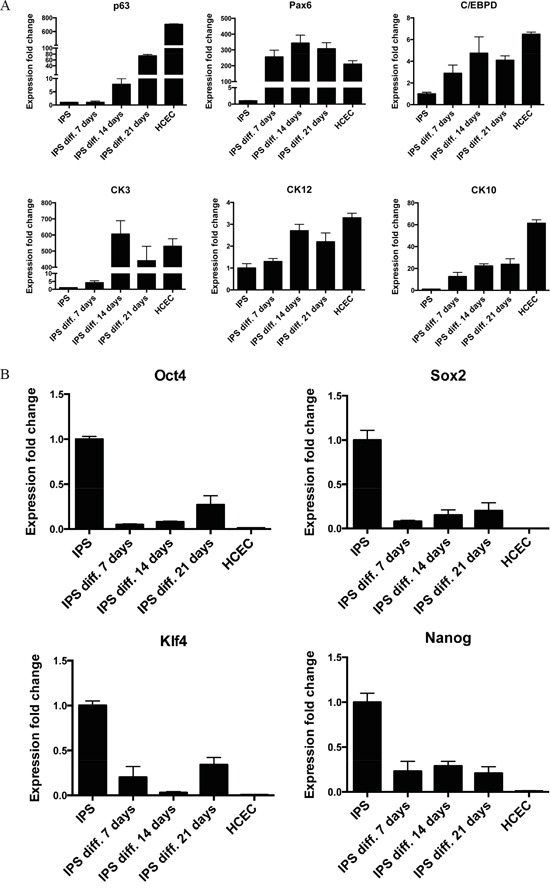
RT-qPCR confirmation of successful differentiation of iPS cells **A.** RT-qPCR analysis for corneal epithelial markers ΔNp63, Pax6, C/EBPδ, CK3, CK12, and the skin epithelial marker CK10. **B.** RT-qPCR analysis for pluripotency markers Oct4, Sox2, Klf4 and Nanog. Cells were analyzed on day 7, day 14, and day 21 of differentiation. HCEC were used as positive control. The data, presented as fold change (compared to iPS cells), was calculated from the ΔΔCT values by the formula 2^−ΔΔCT^ and it is presented as the mean ± SD from triplicate measurements and 2–5 independent experiments data. Abbreviations: IPS diff. 7, 14, 21 days, differentiated iPS cells after 7, 14 or 21 days; HCEC, human corneal epithelial cells.

To confirm the results from immunocytochemistry a reverse transcriptase qPCR was done to detect the expression level of the stemness markers, and limbal cell markers. Interestingly, contrary to the immunostaining results, the gene expression for ΔNp63 was continuously increasing over the 21 day culture period and Pax6, which could almost not be detected by immunocytochemistry, had a very strong positive fold change of over 250 at all the three time points compared to iPS cells. The expression levels of CK3 and CK12 were increasing over the time of differentiation and reached similar levels of gene expression as HCEC cells. The expression levels for CK10 were increasing over time, but were still below the expression level of HCEC (Figure 5A). No change was observed in the expressions of TCF4, ABCG2 and connexin 43 genes (data not shown).

The immunostaining showed a strong protein expression for all pluripotency markers, RT-qPCR showed a decrease for Oct4, Sox2, Klf4 and Nanog at the mRNA level, though the level of the positive control HCEC, was far lower. This may suggest that the loss of stemness was not achieved completely (Figure 5B). Taking together, we conclude that our differentiation method resulted in the generation of corneal epithelial like cells similar to HCEC cell line but with some “traces” of pluripotency, or contamination with iPS.

### Successful transduction of primary fibroblasts with limbal epithelial regulators

**Figure 6:**
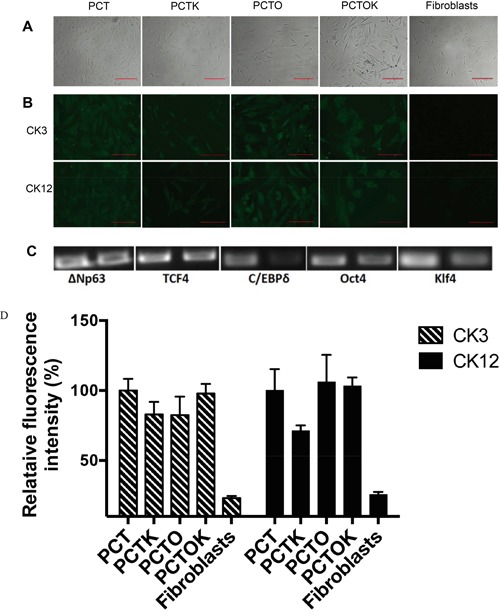
Direct transdifferentiation of human fibroblasts into corneal epithelial lineage **A.** Bright field images of transdifferentiated fibroblasts 7 days post infection either with PCT, PCTK, PCTO or PCTOK (explained in the text). Scale bar: 200 μm. **B.** Immunocytochemistry analysis of transdifferentiated fibroblasts infected with PCT, PCTK, PCTO or PCTOK for the expression of CK3 and CK12. Cells were analyzed on day 7 post infection. Scale bar: 100 μm. **C.** RT-PCR for transduction efficiency analysis. 1^st^ band: transfection of HEK-293 for virus production. 2^nd^ band: transduction of human dermal fibroblasts with virus. Abbreviations: PCT, lentiviral cocktail containing ΔNp63, C/EBPδ and TCF4 lentiviruses; PCTK, lentiviral cocktail containing PCT plus Klf4; PCTO, lentiviral cocktail containing PCT plus Oct4; PCTOK, lentiviral cocktail containing PCT plus Oct4 and Klf4. **D.** Analysis of CK3 and CK12 fluorescence intensities for transdifferentiated fibroblasts infected with PCT, PCTK, PCTO or PCTOK. Results are presented as the percentage of PCT intensity ± SD.

A pool of lentiviruses containing three genes (transcription factors) important in limbal epithelial development (ΔNp63α, TCF4 and C/EBPδ) plus either Oct4 or Klf4 or both (hereafter referred to as PCT, PCTO, PCTK and PCTOK, respectively) was prepared to infect human dermal fibroblasts. To enhance the direct transdifferentiation towards the limbal epithelial lineage the cell medium was changed two days post transduction to a corneal epithelial medium and another two days later cells were seeded on gelatin. Cell morphology stayed the same over the first 4 days, but just upon re-seeding the infected cells on gelatin-coated wells induced morphological changes (Figure 6A & Supplementary Figure S2). The cells developed a more triangular, cobblestone appearance typical for corneal epithelial cells, although the culture stayed heterogeneous with cells displaying also a more fibroblast-like phenotype. Surprisingly, from the seven different conditions tested (summarized in Table 2), cells infected with only the three corneal developmental transcription factors (PCT) showed the highest degree of morphological changes and proliferated much faster than cells subjected to other conditions (images have been quantified using Image J software and the confluency of cells after 14 days has been scored: PCT- 95%, PCTK- 17%, PCTO- 61% PCTOK- 6%). When either Oct4 or Klf4 was added to the three corneal developmental transcription factors, cells also started to change morphology but more cells kept their original fibroblast-like appearance.

**Table 2:** Summary of direct transdifferentiation results

	PCTOK	PCTK	PCTO	PCT	PC	PT	CT
**Morphological changes**	+	+	++	++	-	-	-
**CK3**	++	+	++	++	-	-	-
**CK12**	++	+	++	++	-	-	-

Cells transduced with all five factors (PCTOK) showed a high degree of cellular stress with lots of apoptosis and even after 14 days in culture (Supplementary Figure S3) those cells still displayed a slower cell proliferation and less morphological changes. Cells transduced with only two factors (PC, PT and CT) did not show any change in the morphology (Table 2).

To further assess the degree of transdifferentiation, the infected cells were stained 7 days post infection, for CK3 and CK12 (Figure 6B, 6D). Additionally reverse transcriptase PCR clearly showed that all 5 transcription factors that were introduced into the host's genome, have been expressed at day 3 post infection (Figure 6C). The staining for CK3 and CK12 was positive for four conditions (no staining for cells treated with only two factors was observed), though infection with PCTK showed a little bit weaker expression compared to the other three conditions (Figure 6B, 6D). Pax6 protein was not detected in either of the conditions (data not shown). These results suggest that transduction of fibroblasts with transcription factors important for corneal epithelial development induced changes to a more corneal epithelial-like cell type.

**Figure 7:**
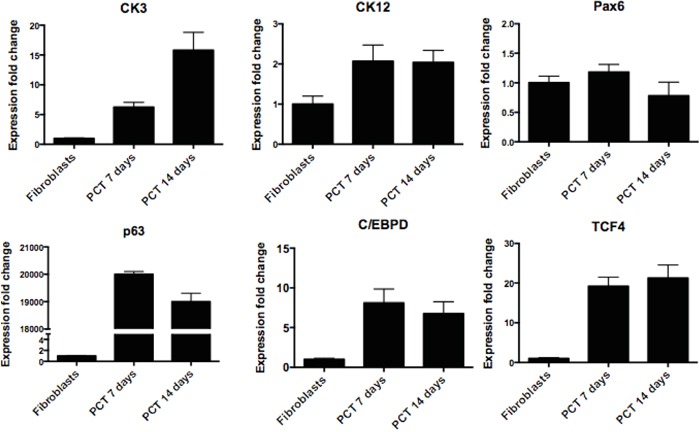
RT-qPCR confirmation of successful direct transdifferentiation of human fibroblasts into corneal epithelial lineage RT-qPCR analysis for corneal epithelial markers CK3, CK12, Pax6, ΔNp63, C/EBPδ and TCF4. The data, presented as fold change (compared to fibroblasts), was calculated from the ΔΔCT values by the formula 2^−ΔΔCT^ and it is presented as the mean ± SD from triplicate measurements and 2–5 independent experiments data. Abbreviations: PCT 7 days, PCT 14 days-fibroblasts transdifferentiated with lentiviral cocktail containing ΔNp63, C/EBPδ and TCF4 after respectively 7 and 14 days.

To check if the proposed transdifferentiation protocol also results in the elevation of the expression of genes specific for limbal epithelial cells, we performed qPCR analysis (Figure 7). We investigated the gene expression only under the condition where fibroblasts were transduced with only three transcription factors (TCP) because such condition achieved the highest degree of transdifferentiation. The expression levels of CK3 and CK12 genes were increasing over the time and reached ~16 and 2.1 fold increase respectively when compared with untreated fibroblasts. Very high expressions were observed for ΔNp63α, C/EBPδ and TCF4, however it has to be remembered that these genes had been introduced with the help of lentiviral vectors. No change in the expression of Pax6 transcript was observed.

## DISCUSSION

In this study we performed the transdifferentiation of human dermal fibroblasts into limbal cells, and compared the efficiency of the process to differentiation of limbal cells from iPS (reprogrammed from human dermal fibroblasts). Since transdifferentiation of human epithelial cells into human limbal cells has not been done before, we had to test several different conditions and various combinations of transcription factors, to achieve the conversion.

The pioneering work by Yamanaka and colleagues to induce pluripotent cells from somatic cells by retroviral infection of defined transcription factors in 2006 [8], paved the way for others to improve the protocols, using different approaches for generating iPS cells, amongst others lentiviral- [13] and adenoviral-delivery systems [17] as well as non-viral systems using expression plasmids [18–20] and transposons [21]. Another strategy is the use of small molecule compounds like the DNA methyltransferase inhibitor 5′-azacytidine and the histone deacetylase inhibitor valproic acid [22]. We used a retroviral-delivery system for introducing the four transcription factors into dermal fibroblasts as well as valproic acid and hypoxia conditions to increase reprogramming efficiency. In addition, we added the ROCK inhibitor Y-27632 that had been shown to enhance the survival and growth of ES cells under unfavorable conditions such as dissociation [23]. This reprogramming approach yielded a good efficiency with colonies showing morphology similar to ES-cells and expression of pluripotency markers. Furthermore, our iPS cells have been expanded over more than 15 passages without any loss of ES-cells typical morphology, thus further indicating the successful reprogramming of fibroblasts into iPS cells. The cell cycle of our iPS cells has also been previously characterized [24]. The analysis has confirmed short G1 phase and high number of cells in G2 phase, which is in accordance with other reports describing iPS cells.

Adult stem cells reside within specialized microenvironments in which they are kept undifferentiated due to secreted signals from surrounding cells, cell-cell interactions and adhesion to extracellular matrix components [25, 26]. As stem cells divide into more differentiated cells they migrate out of this niche and have access to new cues from the environment, which leads to further differentiation steps [27]. The properties of the microenvironment, including the composition of available on the surface chemical groups, its elasticity, even movement, may affect cell differentiation [28]. Hence, changed environment (i.e. changed culture conditions) will prompt to differentiate iPS cells into certain lineages. Using collagen type IV as coating material and a cell culture medium specific for the growth of keratinocytes in 2011 Sakurai and colleagues succeeded for the first time in differentiating mouse iPS cells into stratified epithelial progenitor cells [29]. One year later Hayashi et al. succeeded in inducing corneal epithelial cells from human iPS cells via the SDIA method. This method utilizes a feeder layer and a differentiation medium [30]. Consequently, several groups followed similar protocols, most of them used Collagen type IV as coating material as this is the most abundant basement membrane component of the cornea [31–33]. Collagen type IV promotes the proliferation of p63-positive cells, a marker for corneal epithelial cells [34], though it is quite expensive. We have recently shown that LESCs could be derived from human pluripotent stem cells under serum-free conditions by growing them on commercially-available bioengineered collagen matrices (LinkCell™) mainly comprised of medical grade collagen type I [35]. These results show that LESCs derived from iPS and from embryonic stem cells proliferate 2-4 folds better on the bioengineered type I collagen matrices than on the collagen IV-coated control petri dishes. Recently, the importance of autophagy in epidermal development has been described, which may suggest possible future solutions for enhancing epithelial cell differentiation and transdifferentiation, for instance, by targeting autophagy related pathways [36]. As autophagy profoundly affects the metabolism of not only epithelial cells but also other cell types [37–39], hence autophagy modulators should be tested also as modulators of transdifferentiation.

Here we show that iPS cells could be rapidly differentiated into corneal epithelial cells by seeding them on gelatin-coated culture dishes instead of collagen type IV-coated dishes, and by using conditioned corneal epithelial medium. Gelatin consists of denatured collagen polypeptide chains, which were isolated from bovine or porcine skin or bone by acid or base extraction. Due to this isolation procedure lot-to-lot variance is an issue [40]. We used gelatin from porcine skin and were able to successfully induce the differentiation, though no comparison was made with collagen type IV coating. To reduce the lot-to-lot variance and other issues related with using products from animal origin, recombinant gelatin could be used.

Our differentiation protocol led to changes in morphology as well as expression of cornea epithelial specific markers. CK3 and CK12 are key markers for terminally differentiated corneal epithelial cells, whereas ΔNp63α is a proposed marker for limbal stem cells [3]. Our differentiated cells express all three markers and their protein expression seemed to stable over the 21-day culture period. Another marker for ocular and neural tissue is Pax6 which is expressed in the developing eye and continues to be expressed in the mature corneal epithelium [41]. Surprisingly, we could detect only weak protein expression of Pax6 in the differentiated cells for all the time points tested; similarly, the human corneal epithelial cells (positive control) did not show high levels of protein expression for Pax6 despite the fact that RT-qPCR results suggested the opposite, e.g., the increase in gene expression for Pax6 was at least 200–fold more than undifferentiated iPS cells, similar to those for HCEC. The observed discrepancy may be due to the low affinity of the antibody used for Pax6 detection. Also, gene expression levels for ΔNp63α increased towards the end of the 3-week culture period. The differences in the results obtained by the two methods may be attributed to the post-transcriptional regulatory processes (i.e. miRNAs) regulating the translatory potential of mRNA. Hence, mRNA may be degraded before it is translated into proteins, therefore, the correlation between protein levels and abundance of corresponding mRNA is not very strong [42].

The formation of teratomas upon transplantation of iPS cells into immunosuppressed mice is one of the hallmarks of pluripotent cells. This feature limits the use of iPS cells in the clinic, as it must be ensured that differentiated cells do not possess pluripotency prior to transplantation. Therefore, we tested the differentiated cells for Oct4, Klf4, Sox2, and Nanog expression. Immunostaining revealed that the differentiated cells did not completely lose their pluripotency, as most of these markers were still expressed. RT-qPCR results show the opposite, all genes were down-regulated in the differentiated cells, however, the expressions of Oct4 and Klf4 increased after 21 days of differentiation process. Klf4 is important for postmitotic epithelial cells, especially skin, gut [43] and corneal epithelial cells [44]. This phenomenon explains why differentiated cells show quite similar mRNA of KLF4 levels as iPS cells. One possible reason of such increase in expression could be the reactivation of the transgene. Normally, the transgenes are expressed in the beginning of the reprogramming event and once stable iPS cells are generated, the transgenes are silenced and only the endogenous genes are solely expressed [9]. But it was shown for c-myc that the transgene could get reactivated and consequently drive tumor formation [45]. As Klf4 is a proto-oncogene, reactivation of the transgene could lead to the formation of teratomas and possibly other types of tumors, when transplanted. Therefore, further tests are necessary to reveal if the transgene was reactivated.

As for the generation of iPS cells with defined factors, cells can be directly reprogrammed into another cell type without dedifferentiation to a pluripotent state by introducing tissue specific transcription factors [46]. This was successfully shown for transdifferentiating fibroblasts into the neural lineage [15] and cardiomyocytes [16]. For transdifferentiating fibroblasts into the corneal epithelial lineage no protocol exists so far. Thus, we set out to determine if the introduction of key developmental limbal epithelial transcription factors could transdifferentiate human fibroblasts into the corneal epithelial lineage. ΔNp63α, C/EBPδ and TCF4 are important developmental limbal epithelial regulators, where ΔNp63α seems to play a role in the proliferative potential of limbal epithelial cells as it is solely expressed by stem-like cells and not terminally differentiated cells [47]. C/EBPδ is responsible for the self-renewal of limbal epithelial stem cells by forcing the cells into the G_0_/G_1_ phase of the cell cycle and keeping them quiescent [48]. TCF4 maintains the functional properties of corneal epithelial stem cells, is exclusively found in the limbal epithelium and co-localizes with ΔNp63α [49]. To enhance the transdifferentiation potential the cells were also infected with Oct4 or Klf4. Those two pluripotency markers were chosen due to their expression in either limbal stem cells (Oct4) [50] or corneal epithelial cells (Klf4) [44]. The infection of fibroblasts with the cocktail of transcription factors plus changing the medium to a corneal epithelial medium seemed to be sufficient to induce limbal epithelial/corneal epithelial cells, since just upon seeding them on gelatin-coated wells virus transduced cells started to change morphology. Interestingly, cells that were infected with PCT showed the same strong expression of CK3 and CK12 as cells infected with PCTO, PCTK or PCTOK, however PCT cells proliferated and differentiated much faster than cells transduced with four or five factors. Since CK3 and CK12 are associated with terminally differentiated cells, a strong staining indicates successful change towards corneal epithelial cell state. As we did not observe any changes in cells treated with combinations of only two factors, it suggests that at least three of them are required for successful transdifferentiation. Nevertheless, all culture conditions showed a heterogeneous cell population, with many cells similar to corneal epithelial cells and some cells displaying a more fibroblast-like state. Taken together, these results indicate a direct conversion of fibroblasts to the corneal epithelial lineage is possible, but to various degree, and thus the protocol has to be further refined to produce pure corneal epithelial-like cells.

### Conclusion

The present study shows that corneal epithelial cells could be generated by differentiating human iPS cells with the help of gelatin-coated surfaces and corneal epithelial conditioned medium. Our differentiation strategy results in a rapid change of morphology to induce complete differentiation in 14-21 days. Furthermore, corneal epithelial-like cells could be generated by introducing defined factors into human fibroblasts. This is a first step towards generating corneal epithelial cells by transdifferentiation (direct conversion), however several improvements to the protocol still need to be done to assure a full, uniform differentiation, as well as further tests to assess the functional properties of the generated cells.

## MATERIALS AND METHODS

### Cell culture

HEK293, human normal dermal fibroblasts (obtained as waste-product from cosmetic surgery and provided by Dr. Gunnar Kratz [51]), and limbal stromal cells (provided by Dr. May Griffith) were cultured in DMEM medium (PAA, Pasching, Austria) with 10% FBS (PAA) and 1% penicillin-streptomycin (GIBCO, Carlsbad, CA) antibiotics. Human corneal epithelial cell line (HCEC) (provided by Dr. May Griffith) was cultured in EpiLife medium (GIBCO) with 1% human corneal growth supplement (HCGS; GIBCO). Human iPS cells were maintained on mitomycin-C treated MEF feeder cells in primate ES cell medium (ReproCell, Yokohama, Japan). Cultures were kept at 37°C, 5% CO_2_ and 21% O_2_. All cell lines were maintained at a confluence of ~70%.

### IPS cell generation

IPS cells were generated by infecting human dermal fibroblasts with Oct4, Klf4, c-myc and Sox2 [24]. Retroviruses containing the 4 reprogramming factors were independently produced by transfecting HEK293 cells using polyethylenimine (PEI, Polysciences Inc., Warrington, PA) based method, with vectors containing human Oct4, c-myc, Sox2 and Klf4, gag-pol plasmid pUMVC and pCMV-VSV-G envelope plasmid in DMEM containing 10% FBS. 48, 72 and 96 h after transfection viral supernatants were collected, filtered through 0.45 μm low protein binding PVDF filters (Millipore, Billerica, MA) and concentrated by centrifugation at 4000g for 1 h at 4°C using Amicon^®^ Ultra Centrifugal Filter Devices (Millipore). For reprogramming 50,000 fibroblasts/well were seeded in 6-well plates and infected on the next day with the mixture of viral concentrates in presence of 8 μg/ml polybrene (Sigma-Aldrich, St. Louise, MO). Transduced fibroblasts were kept in DMEM medium containing 1 mM valproic acid (Sigma-Aldrich) and 10 μM ROCK inhibitor (Y-27632; Wako reagent, Osaka, Japan) for 6 days. Then, cells were treated with trypsin/EDTA (PAA) and seeded on mitomycin-C treated MEF cells (5.5 × 10^4^ cells/cm^2^) in Primate ES Cell Medium supplemented with 1 mM valproic acid and 10 μM ROCK inhibitor. Cells were kept under hypoxic conditions (5% O_2_) with daily medium change. After 2 weeks first round ES-cell like colonies with sharp edges could be detected. To establish iPS cell lines, iPS colonies were picked up enzymatically using 1 mg/ml Collagenase/Dispase (Roche, Basel, Switzerland), dissociated mechanically into smaller clumps and seeded in Primate ES Cell Medium onto fresh mitomycin-C treated MEF cells and cultured under standard cell culture conditions.

### Conditioning of corneal epithelial medium by limbal stromal cells

Corneal epithelial medium was conditioned as previously described [34]. In brief, confluent limbal stromal cells were mitotically inactivated by incubation with 10 μg/ml mitomycin-C (Abcam) in DMEM medium for 3 h at 37°C. Cells were washed 2 times with PBS and EpiLife basal medium containing 1% human corneal growth supplement was added. The limbal stromal-conditioned medium was collected daily and replaced with fresh corneal epithelial medium for 10 days. The conditioned medium was sterile filtered using a 0.22 μm -filter and stored at −20°C until use for IPS cell differentiation.

### Corneal epithelial differentiation of human IPS cells

iPS cells were harvested using 1 mg/ml Collagenase/Dispase, washed with PBS, mechanically dissociated into smaller clumps and seeded on 0.1% gelatin-coated 6-well plates. Cells were grown in limbal stromal-conditioned medium with medium changes every 2-3 days. Cells were dissociated using Trypsin/EDTA and reseeded on gelatin-coated 6-well plates. Differentiation was observed over a 2-day period. For immunocytochemistry assays cells were trypsinized and seeded on 24-well plates coated with 0.1 % gelatin on day 7, 14 and 21. At the same time points RNA was extracted.

### Transdifferentiation of human dermal fibroblasts into limbal epithelial lineage

Lentiviruses containing the transdifferentiation factors were independently produced by transfecting HEK293 cells (PEI) with vectors containing human ΔNp63α (kindly provided by Dr. Wendy Weinberg), TCF4, C/EBPδ (abmGood), Oct4 and Klf4, gag-pol plasmid pCMVΔR8.91 and VSV-G envelope plasmid pMDG in DMEM medium. 48, 72 and 96 h after transfection viral supernatants were collected, filtered through 0.45 μm low protein binding PVDF filters and concentrated by centrifugation at 4000 g for 1 h at 4°C using Amicon^®^ Ultra Centrifugal Filter Devices. For transdifferentiation 50,000 human dermal fibroblasts/well were seeded in 6-well plates in FibroGRO™ complete medium (Millipore) and infected on the next day with a mixture of viral concentrates (either without Klf4 or Oct4, with Klf4 or Oct4, or both) in the presence of 8 μg/ml polybrene. Two days post infection the medium was changed to EpiLife basal medium containing 1% human corneal growth supplements. 4 days post infection cells were seeded onto 0.1% gelatin-coated wells and morphological changes were observed. For the immunocytochemical analysis, cells were seeded in 24-well plates and analyzed on day 7 post infection.

### Immunocytochemistry

The Immunocytochemistry has been performed similarly as described previously [52]. The medium in the tissue culture well was removed and cells were fixed with 4% PFA (Santa Cruz Biotechnology, Dallas, TX) for 20 min, permeabilized with 0.2% Triton X-100 (Sigma-Aldrich) for 20 min, blocked in 1% BSA (Sigma-Aldrich) for 1 h and incubated with primary antibody diluted in blocking solution (dilution 1:100 except anti-hSox2 dilution 1:20) overnight at 4°C. Cells were stained with their respective secondary antibody with conjugated fluorophore diluted in blocking solution for 1 h, counterstained with DAPI and treated with mounting medium. All incubation steps were done at room temperature except otherwise stated and in-between washing steps were conducted using PBS. To control for unspecific binding a negative control with just secondary antibody was done. Stained cells were observed using fluorescent microscopy (AxioVert.A1, Carl Zeiss Jena GmBH, Jena, Germany) and edited using ZEN imaging software. The fluorescence intensities were quantified using ImageJ software (available at http://rsb.info.nih.gov/ij; developed by Wayne Rasband, NIH).

### Primary antibodies used in this study

Rabbit anti-Oct4, mouse anti-Klf4, rabbit anti-Nanog (all Stemgent, Cambridge, MA), goat anti-hSox2 (R&D Systems, Minneapolis, MN), goat anti-ΔNp63 (N-16), rabbit anti-C/EBPδ (M-17), mouse anti-ITF-2 (C-1), goat anti-K12 (N-16), goat anti-K3 (Q-14) (all Santa Cruz Biotechnology), rabbit anti-c-myc, mouse anti-Pax6 (both Abcam, Cambridge, UK), mouse anti-ABCG2 (Abcam, Cambridge, UK). Secondary antibodies used were: Alexa-Fluor^®^ 488 goat anti-rabbit IgG (Molecular Probes, Eugene, OR), Alexa-Fluor^®^ 488 donkey anti-goat IgG (Abcam), FITC goat anti-mouse IgG (Sigma-Aldrich).

### RNA isolation, cDNA synthesis, RT-PCR & quantitative RT-PCR

The procedure was performed similarly as described previously [53]. Total RNA was obtained using the High Pure RNA Isolation Kit (Roche). Reverse transcription was performed using the Maxima^®^ First Strand cDNA Synthesis Kit for RT-qPCR (Fermentas, Burlington, Canada) according to the manufacturer's suggested protocol, and cDNA was used as a template for PCR or stored at −20°C. To check the successful infection of transgenes RT-PCR was performed using DreamTaq Green PCR Master Mix (Fermentas) and then subjected to 1.5% agarose gel electrophoresis. Quantitative RT-PCR was performed in the CFX96™ Real-time PCR system (BioRad, Hercules, CA) with the iQ™ SYBR^®^ Green Supermix (Bio-Rad) using specific primers (final concentration 0.5 μM; Invitrogen, Carlsbad, CA) listed in Table 3. As an internal reference glucose-6-phosphate dehydrogenase (GAPDH) was used and undifferentiated iPS cells as calibrator to determine the relative quantities of gene expression in each sample. The thermocycling program was performed as follows: 1 cycle at 95°C for 3 min and 40 cycles at 95°C for 15 s and 45 s at primer-specific annealing/extension temperature, followed by a melt curve analysis to control the quality of all PCR products (no nonspecific amplification or primer-dimer was observed in any of the reactions.). Cycle threshold (C_T_) values were obtained from the logarithmic amplification phase and the relative quantification of each gene was calculated applying the 2^−ΔΔCt^ method [54]. All assays were run in triplicates of 3-5 biological samples for each time point of differentiation.

**Table 3:** Specification of primers used for RT-qPCR

Gene		Primer sequence	Annealing/Extension temperature (°C)
Oct4	forward	TCTCGCCCCCTCCAGGT	60
	reverse	GCCCCACTCCAACCTGG	
Klf4	forward	CCGCTCCATTACCAAGAGCT	59
	reverse	ATCGTCTTCCCCTCTTTGGC	
Sox2	forward	CGAGTGGAAACTTTTGTCGGA	59
	reverse	TGTGCAGCGCTCGCAG	
Nanog	forward	AGAAGGCCTCAGCACCTAC	57
	reverse	GGCCTGATTGTTCCAGGATT	
ΔNp63	forward	CTGGAAAACAATGCCCAGAC	59
	reverse	GGGTGATGGAGAGAGAGCAT	
CK3	forward	TTAAGGACCCTCTACGACGC	59
	reverse	AATGATGCTGTCCAGGTCCA	
CK12	forward	TGGAGATTGAGACCTACCGC	59
	reverse	ACCATTCACCATCTCCTGCA	
Pax6	forward	ATAACCTGCCTATGCAACCC	60
	reverse	GGAACTTGAACTGGAACTGAC	
C/EBPδ	forward	CCATGTACGACGACGAGA	60
	reverse	GCCTTGTGATTGCTGTTGAAGA	
TCF4	forward	GTAGTGCCATGGAGGTACAGAC	60
	reverse	TGTCTGCTGAGGAGTGTGATG	
CK10	forward	GGCTGACCTGGAGATGCAAAT	60
	reverse	GGGGCAGCATTCATTTCCACA	
ABCG2	forward	GTGCACATGCTTGGTGGTCTTGTT	60
	reverse	AGCTCGGTCTTAACCAAAGGCTCA	
Connexin 43	forward	GCGTGAGGAAAGTACCAAAC	60
	reverse	GGGCAACCTTGAGTTCTTCC	
GAPDH	forward	GTCAGTGGTGGACCTGACCT	57-60
	reverse	CACCACCCTGTTGCTGTAGC	

### Statistical analysis

Analysis of RT-qPCR was done in Microsoft Office Excel. All values are represented as mean ± SD.

## SUPPLEMENTARY FIGURES



## Abbreviations

ABCG2: ATP-binding cassette sub-family G member 2
C/EBPδ: gene for CCAAT/enhancer binding protein (C/EBP), delta
CEBPD: CCAAT/enhancer binding protein (C/EBP), delta
CK3: cytokeratin 3
CK10: cytokeratin 10
CK12: cytokeratin 12
*c-myc*: a gene for c-Myc oncogenic transcription factor
C_T_: cycle threshold
DAPI: 4′6-diamidino-2-phenylindol
EDTA: ethylenediaminetetraacetic acid
ES: embryonic stem cells
FBS: fetal bovine serum
GAPDH: glucose-6-phosphate dehydrogenase
HCEC: human corneal epithelial cells
HCGS: human corneal growth supplement
HEK293: human normal dermal fibroblasts
iPS: induced pluripotent stem cells
iPS diff.: differentiated iPS cells
ITF2: immunoglobulin transcription factor 2; basic helix-loop-helix protein
*Klf4*: Kruppel-like factor 4 (gut); transcription factor
LESCs: limbal epithelial stem cells
Lin28: lin-28 homolog A; gene encodes LIN-28 family RNA-binding protein
LSC: limbal stem cell
MEFs: mouse embryonic fibroblasts
miRNAs: micro RNA, a small non-coding RNA molecule
mitomycin C: {11-Amino-7-methoxy-12-methyl-10,13-dioxo-2,5-diazatetracyclo[7.4.0.0^2,7^.0^4,6^]trideca-1(9),11-dien-8-yl}methyl carbamate; an anticancer drug
Nanog: nanog homeobox, a DNA binding homeobox transcription factor involved in embryonic stem cells (ES) proliferation, reneval and pluripotency
*Oct4*: octamer-binding transcription factor 4
Pax6: paired box gene 6
PBS: phosphate-buffered saline
PCT: lentiviral cocktail containing ΔNp63, C/EBPδ and TCF4 lentiviruses
PCTK: lentiviral cocktail containing PCT plus Klf4
PCTO: lentiviral cocktail containing PCT plus Oct4
PCTOK: lentiviral cocktail containing PCT plus Oct4 and Klf4
PEI: polyethylenimine
PFA: paraformaldehyde
PVDF filters: polyvinylidene fluoride filters
quantitative RT-PCR: real time quantitative reverse transcription polymerase chain reaction
RT-PCR: reverse transcription polymerase chain reaction
*Sox2*: transcription factor, also known as SRY –box2 (sex determining region Y)-box 2
TCF4: transcription factor 4 (immunoglobulin transcription factor 2; ITF-2)
ΔNp63α: unique C-termini α isoform of tumor protein p63.
