# Supplementary material for: Human induced pluripotent stem cell differentiation and direct transdifferentiation into corneal epithelial-like cells
Source: Oncotarget. 2016 Jun 2;7(27):42314–29. doi: 10.18632/oncotarget.9791 (PMC5173137; doi:10.18632/oncotarget.9791)
Supplement: Supplementary file 1 [file oncotarget-07-42314-s001.pdf]

Human induced pluripotent stem cell differentiation and direct transdifferentiation into corneal epithelial-like cells

SUPPLEMENTARY FIGURES

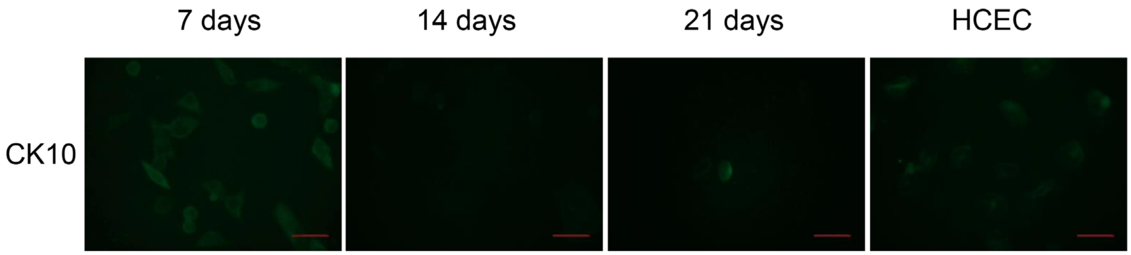

Supplementary Figure S1: Differentiation of iPS cells to the corneal epithelial lineage. Immunocytochemical analysis for expression of CK10 at day 7, day 14 and day 21. Scale bar: 50 µm.

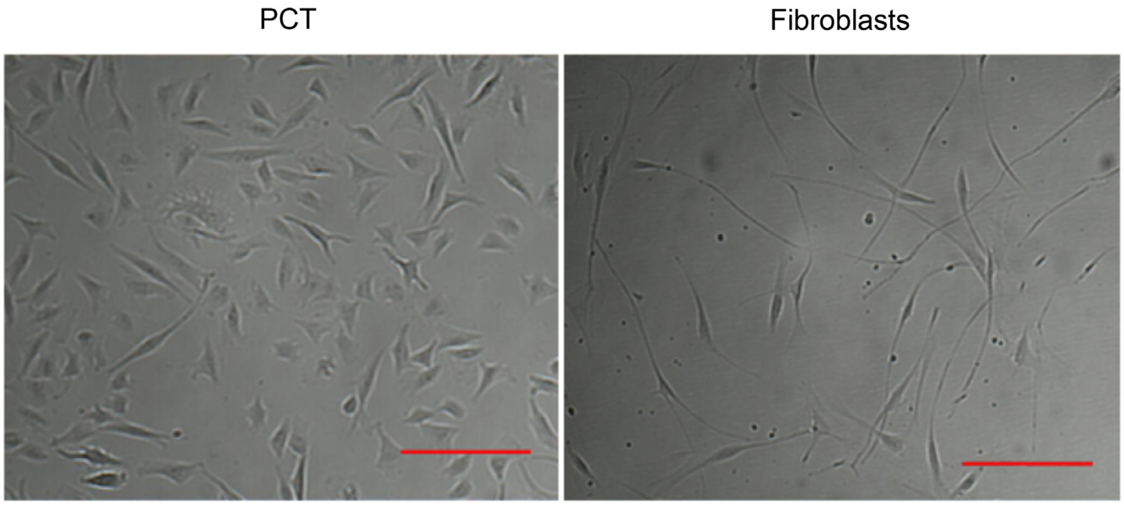

Supplementary Figure S2: Comparison between untreated fibroblasts and fibroblasts directly transdifferentiated into corneal epithelial lineage. Bright field images of transdifferentiated cells 7 days post infection with PCT and untreated fibroblasts. Scale bar: 200 µm.

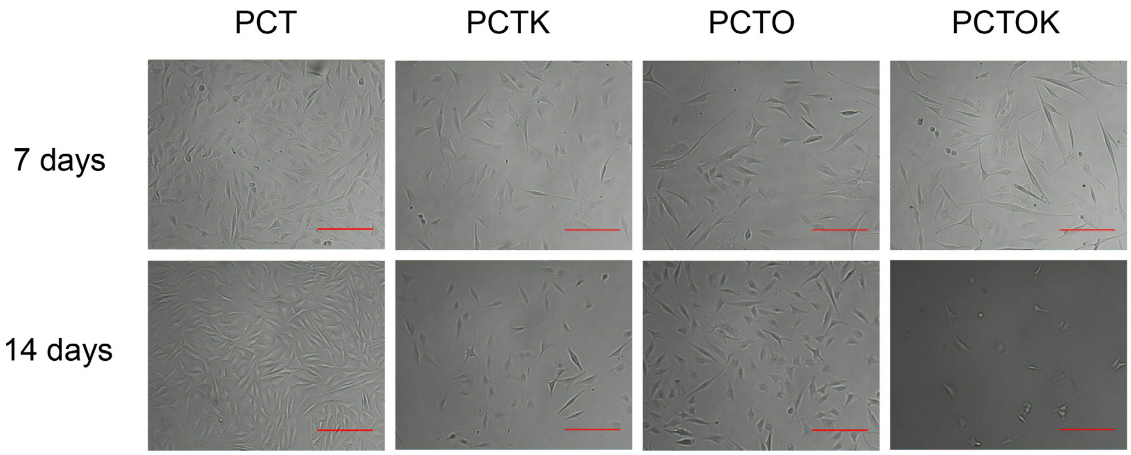

Supplementary Figure S3: Fibroblasts directly transdifferentiated into corneal epithelial lineage for 7 and 14 days. Bright field images of transdifferentiated fibroblasts 7 and 14 days post infection either with PCT, PCTK, PCTO or PCTOK. Scale bar: 200 μm.
